# Evaluation of Histopathological Changes in Control Biopsies Which Taken 48 Sessions after NBUVB Phototherapy for Early-Stage Mycosis Fungoides

**DOI:** 10.1100/2012/426732

**Published:** 2012-09-02

**Authors:** Ebru Zemheri, Seyma Ozkanli, Ilkin Zindanci, Serkan Senol, Ozge Akbulak, Elvan Turfanda, Mehtap Toprak, Duygu Kosemetin, Abdullah Aydin

**Affiliations:** ^1^Department of Pathology, Training and Research Hospital, Istanbul Medeniyet University, SB Goztepe, Turkey; ^2^Department of Dermatology, Training and Research Hospital, Istanbul Medeniyet University, SB Goztepe, Turkey

## Abstract

*Background.* There are not many studies investigating histomorphological changes in 48 sessions in patients with early-stage MF after narrowband UVB (NBUVB) treatment. Our purpose is to evaluate histological features of phototherapy after 48 sessions and determine which parameters are more reliable for controlling skin biopsies. *Methods.* Biopsies of 32 patients with early stage of MF, who were treated with NBUVB phototherapy, were histologically evaluated before and after the treatments, including epidermotropism, stratum corneum, epidermal thickness, dermal infiltration, papillary dermal fibrosis, vascular alterations, and other dermal changes. We discuss the histomorphological effects of NBUVB phototherapy on skin biopsies by comparing the responders with nonresponders, with before and after the treatment. *Results.* 9 patients (28%) did not give any response to treatment. Alleviation in epidermotropism, increases in parakeratosis and normal keratosis, perivascular infiltration, and melanophages, decrease in the lichenoid/patchy lichenoid infiltration pattern after the treatment was statistically significant. Comparing by response, normalization of stratum corneum and epidermis, orthohyperkeratosis, decrease in linearly arranged cells, the lichenoid/patchy lichenoid infiltration, the loss of inflammation were statistically significant in responders group. *Conclusion.* We detected a significant decrease in linearly arranged cells after phototherapy, indicating that it is an “important diagnostic parameter" in evaluation of therapeutic response.

## 1. Introduction

 Mycosis fungoides (MF) is the most common form of cutaneous T-cell lymphoma. MF lesions are divided into three stages: patch, plaque, and tumor [1, 2]. Early-stage (stages IA, IB, and IIA) MF has long been treated with various agents including topical potent corticosteroids, topical nitrogen mustard, topical carmustine, oral psoralen plus UVA (PUVA), broadband and narrowband UVB, electron-beam radiotherapy, interferon, retinoids, and topical bexarotene [3–5].

Despite a lot of clinical observation, the histopathological changes seen in 48 sessions after NBUVB phototherapy for early-stage mycosis fungoides are not clearly described. So, we compared the histopathological changes in biopsies taken before and after the treatment with NBUVB phototherapy for 48 sessions.

In this study, histopathologic findings between responders and nonresponders to treatment in addition to histopathologic changes before and after the treatment were compared.

## 2. Materials and Methods

### 2.1. Patients

A total of 32 patients with early-stage MF were recruited between 2008 and 2012. All patients were treated with NBUVB phototherapy. We reevaluated histological findings in biopsies taken from the lesions of patients, which were taken before treatment and after each 48 sessions.

The mean age of the patients (18 women and 14 men) was 58 years and ranged from 30 to 71 years.

Evaluations were made according to clinical and histopathological findings described in World Health Organization-European Organization for Research and Treatment of Cancer Diagnostic Criteria [6]. Twenty-nine lesions were classified as patch stage, and three lesions were classified as plaque-stage MF. stage of disease was based on the TNM classification [7]. Five patients were in stage IB and three patients were stage IIA and the rest were stage IA. According to the classification of Fitzpatrick, the observed skin types were as follows: type III in 8 patients, type I in 4 patients, and type II in the rest. None of the patients had organomegaly. There were no abnormalities in routine biochemical investigations, red or white blood cell counts. Computed tomography scans of thorax and abdomen were normal in all patients. Clinical response was classified as follows: more than 90% reduction in skin lesions was considered as complete response; less than 90% reduction in skin lesions was considered as nonresponse.

### 2.2. Phototherapy

Phototherapy was performed in a phototherapy cabinet (7001 K cabinet, Waldman, Germany) containing 21 UVB lamps (Philips TL-01/100 W) which had radiated light at 311 nm of wavelight. Initial dose was 0.2 joule/cm^2^, and dosage was increased 0.1 joule/cm^2^ in each two séance. Maximum dosage was 2.9 joule/cm. The phototherapy was administered three times a week.

### 2.3. Histopathological Evaluation

We determined histomorphological parameters according to recently published histological criteria for early lesions of MF [1, 7, 8]. Epidermotropism was the presence of atypical lymphoid cells in the epidermis and it was classified as single/haloed lymphocytes, linearly arranged single lymphocytes and Pautrier microabscesses. Stratum corneum was classified as normal, orthohyperkeratotic, and parakeratotic. The epidermal thickness was grouped as normal, atrophic, and hyperplastic. Dermal infiltration pattern was classified as superficial perivascular, lichenoid/patchy lichenoid, and no inflammation. Papillary dermal fibrosis was defined as the degree of increased collagen (0, 1, and 2). Other dermal changes were classified as basal vacuolar degeneration, dyskeratotic cells, and melanophages. Vascular alterations such as telangiectasia and vascular proliferation were evaluated. All parameters were evaluated by two pathologists (E.Z., S.O.).

### 2.4. Statistical Analysis

The data were processed on a personal computer and analyzed using SPSS 15.0 (Statistical Package for Social Sciences) (SSPS Inc., Chicago, IL, USA). Chi-square test, Fisher's Exact Chi-square test, and Mc Nemar test was used for comparison of qualitative data. *P* values < 0.05 were considered statistically significant.

## 3. Results

**Figure 1:**
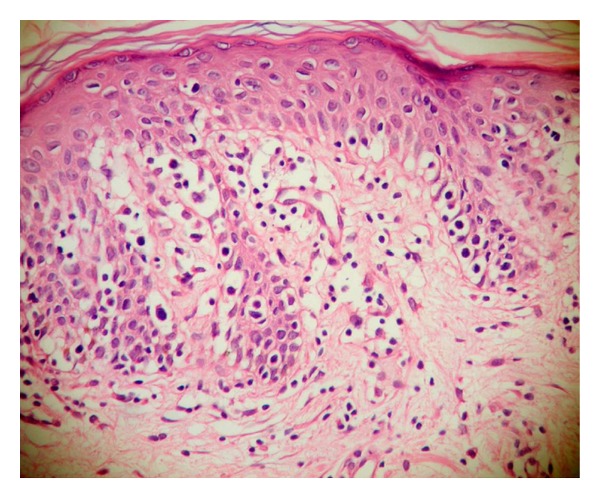
Linearly arranged and single/haloed atypical lymphocytes in epidermis in patient with patch stage MF were seen before treatment (×400, H&E).

**Figure 2:**
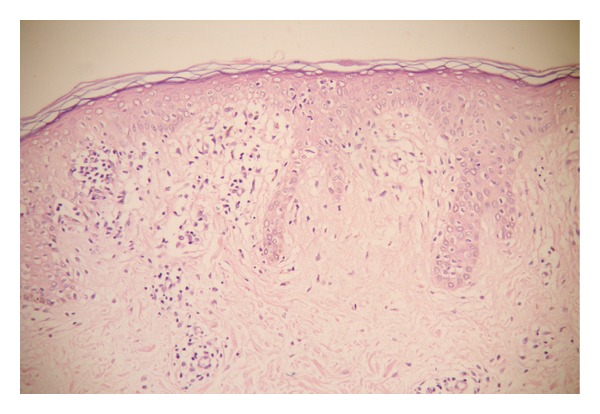
Loss of linearly arranged cells, but persistence of single/haloed lymphocytes and Pautrier microabscess in the epidermis, fibrosis in the papillary dermis in nonresponder group after treatment were seen (×200, H&E).

**Figure 3:**
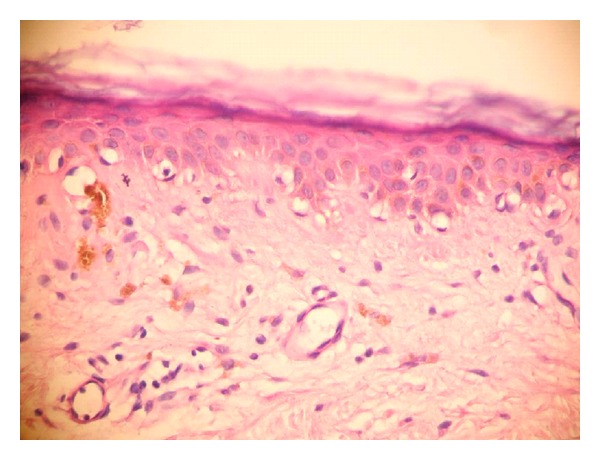
Basal vacuolar degeneration in epidermis and melanophages in papillary dermis were seen after treatment (×400, H&E).

Both clinically and histologically, 9 of 32 patients (28%) did not give any response to treatment, whereas the remaining did. Of nine unresponsive patients, three were at the plaque stage MF and the rest were at the patch stage. Histopathological findings pointing persistence of disease were also present in all patients who were clinically unresponsive to the treatment. The histopathological findings before and after the treatment are listed in Table 1. Before treatment, epidermotropism was established in all cases and linearly arranged cells were the most prominent (93.8%) finding (Figure 1). Single cells and Pautrier microabscesses have followed this finding, respectively (62.5%, 59.4%). Alleviation in all types of epidermotropism after the treatment was found to be highly statistically significant (*P* = 0.001). After the treatment, in seven cases of total, epidermotropism was observed and the single/haloed lymphocytes were the most common type (21.9%) (Figure 2). Although the orthohyperkeratosis in stratum corneum was very prominent before the treatment, parakeratosis (*P* = 0.039) and normal keratosis (*P* = 0.004) in stratum corneum were increased after the treatment. In all cases, inflammation was in lichenoid/patchy lichenoid pattern before the treatment. Following the treatment, decreases in the lichenoid/patchy lichenoid infiltration pattern (*P* = 0.001) and increases in the perivascular infiltration (*P* = 0.001) were noticed, in addition, in 9 cases, there was no sign for inflammation (*P* = 0.004). In evaluation of other changes in dermis and epidermis after the treatment, only the increase in melanophage count was found to be statistically significant (*P* = 0.001) (Figure 3). Vascular changes were not considered as statistically significant.

**Figure 4:**
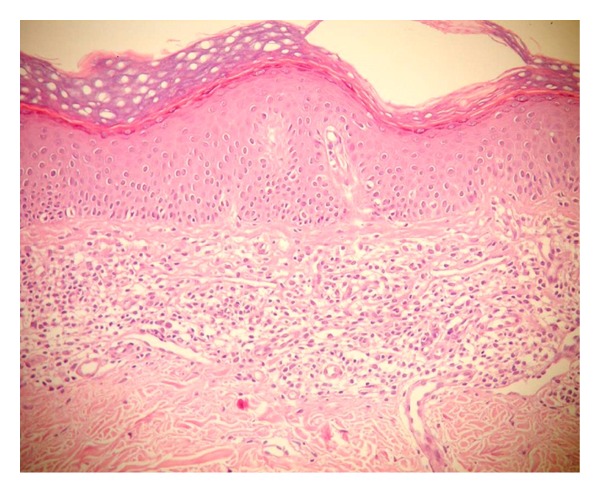
Lichenoid infiltration of atypical cell and fibrosis were seen in nonresponder group after treatment (×200, H&E).

After the treatment, the responders and the nonresponders findings were compared in Table 2, the ratio for observing the linearly arranged cells was found to be significantly lower in the responders (*P* = 0.038). In responders, normalization of stratum corneum was found to be higher compared to the nonresponders (*P* = 0.001), whereas orthohyperkeratosis was found to be lower compared to the nonresponders (*P* = 0.007). In responders, the normalization of epidermis was observed, but it was not statistically significant. On the contrary, in nonresponders, epidermis was significantly hyperplastic (*P* = 0.002). The decrease in the lichenoid/patchy lichenoid infiltration (*P* = 0.004) was found to be highly significant and the cessation of inflammation was also found to be (*P* = 0.027) statistically significant in responders. Of nine nonresponders, 5 had lichenoid/patchy lichenoid pattern (Figure 4), whereas 4 had perivascular pattern. There were atypical lymphocytes in all nine nonresponders. Other epidermal and dermal changes and vascular changes were not statistically significant in both groups (responders and nonresponders).

## 4. Discussion

There are not enough studies investigating histomorphological changes after 48 sessions of NBUVB treatment in patients with early-stage MF.

The main purpose of this study is to evaluate histological features of phototherapy after 48 sessions and to determine which parameters are more reliable for control skin biopsies. We discuss the histomorphological effects of NBUVB phototherapy on skin biopsies by comparing the responders with nonresponders with before and after the treatment.

Early-stage MF may clinically and histologically mimic benign inflammatory dermatoses making it difficult to diagnose [9, 10]. Because of its indolent and chronic course with recurrences and its possibility to progress to the aggressive course (although rare), this disease must be treated as early as possible.

The histological diagnosis of early-stage MF may be difficult in many instances. The histological parameters in diagnosis of MF have been assessed in a number of studies [8, 11–13]. The International Society for Cutaneous Lymphomas (ISCL) and the cutaneous lymphoma task force of the European Organization of Research and Treatment of Cancer (EORTC) revised the staging and classification of mycosis fungoides and Sézary syndrome. In this revision, they reported that making the definitive histopathologic diagnosis with light microscopy alone may be difficult in early MF. The ISCL has recently proposed a diagnostic algorithm for early-stage MF. According to this classification, superficial lymphoid infiltration with epidermotropism without spongiosis and lymphoid atypia which is defined as cells with enlarged hyperchromatic nuclei and irregular or cerebriform nuclear contours are histopathologic criteria for early-stage MF diagnosis [7].

We have taken into consideration epidermotropism in our study for diagnosing MF and in determining the responder group after the treatment. We have detected a significant decrease in linearly arranged cells after phototherapy, indicating that it is an “important diagnostic parameter” in the evaluation of therapeutic response. There was a decrease in single cells and Pautrier microabscesses, although we think that it is less important in evaluating the response to the treatment. Apa et al., in their report, have indicated linearly arranged cells (like in our study) and Pautrier microabscesses as active parameters [1].

When comparing the histological findings of responsive and unresponsive groups, it came into our attention that, in responsive group, inflammation and epidermotropism were attenuated, meanwhile the stratum corneum and epidermis were in normal boundaries. In both groups, reactive changes had similar characteristics. These findings support that the NBUVB not only contributes to the depletion of the epidermotropism, but also contributes to the normalization of the epidermis.

Gökdemir et al. has determined the effects NBUVB in early-stage MF both clinically and histopathologically. Histopathologic response was divided into three categories. First, the complete response, the absence of epidermotropism and Pautrier microabscesses marked the reduction in dense infiltrates comprising atypical lymphocytes with irregular nuclei; second, the partial response, marked the reduction in epidermotropism and sparsely scattered atypical lymphocytes in the epidermis and dermis; third, no response and no histopathologic changes. Of all patients, 18 (78.26%) had complete histopathological improvement and five (21.74%) had partial response or no histopathological response [3].

El-Mofty et al. compared the clinical and histopathologic efficacy of PUVA and NBUVB in the treatment of early-stage MF. Histopathological changes were graded as follows: very good response: only sparse inflammatory infiltration in the dermis; good response: mild epidermotropism, sparse infiltration and nonatypical cells; fair response: epidermotropism, dense band-like infiltration and atypical cells; poor response: epidermotropism, dense and deep dermal infiltration, atypical cells. They have detected that 9 patients of 10 show very good-good response and only one patient show fair-poor response on 48 sessions [14].

Hyperkeratosis, hypergranulosis, variable acanthosis, and epidermal atrophy can be seen in treatment with UV [15]. We have found that parakeratosis is a common finding, as in the study of Apa et al., who reported that it was a distinguishing parameter when present at the time of diagnosis [1]. After the treatment, parakeratosis has disappeared in our study, like in the study of Apa et al. Epidermal hyperplasia seemed to be an important distinguishing parameter and, in our study, it has increased after therapy, especially in the responders.

In our study, no significant change in fibrosis has been achieved before and after the treatment. According to Naraghi et al., papillary dermal fibrosis was a sensitive feature (96%), and it has achieved statistical significance as a discriminating factor [8]. Similar results were reported by Smoller et al. [11] and Ackerman [13]. They had also pointed out that dermal fibrosis was a feature of late atrophic patches or plaques and was not encountered in early patches [11, 13]. But Apa et al. had reported that increase in the amount of dermal fibrosis was the most frequent parameter seen after phototherapy [1]. Since all of our cases were early-stage MF, the degree of alteration in fibrosis was consistent with studies of Naraghi and Ackerman.

Epstein had reported that telangiectatic vessels may be conspicuous [16]. Dermal edema and vasculopathy were neither sensitive nor specific for MF [1, 8]. In our study, vascular changes were not established to have any characteristics for the diagnosis. This finding supports that NBUVB has no significant effect on the vascular structures which are not affected by the disease.

Melanin pigmentation, melanocyte hyperplasia, and pigmentary incontinence can be seen in treatment with UV [17–19]. In addition, UV light triggers apoptosis and leads to epidermal basal cell degeneration resulting from cytoplasmic swelling [20, 21]. We have found an increase in dermal melanophages after the treatment, which can be considered as a therapeutic side effect. After the treatment, absence of these secondary changes in responders and nonresponders supports this opinion.

Based on the data presented here, we think that some histological features, such as epidermotropism, changes of stratum corneum, epidermis, and dermis, can be used in determining the effectiveness of treatment. We have found that epidermotropism of atypical cells were important criteria in order to decide whether the disease was histopathologically present or not, and the secondary changes to NBUVB had no use for this purpose.

## Figures and Tables

**Table 1:** Comparison of histopathological changes before and after the treatment.

	Before treatment	After treatment	*P*
	*n* (%)	*n* (%)	
Epidermotropism			
Single/haloed lymphocytes	20 (62.5%)	7 (21.9%)	0.001**
Linearly arranged	30 (93.8%)	6 (18.8%)	0.001**
lymphocytes			
Pautrier microabscesses	19 (59.4%)	4 (12.5%)	0.001**
Stratum corneum			
Normal	7 (21.9%)	21 (65.6%)	0.004**
Parakeratosis	8 (25.0%)	1 (3.1%)	0.039*
Orthohyperkeratosis	17 (53.1%)	10 (31.3%)	0.143
Epidermis			
Normal	9 (28.1%)	15 (46.9%)	0.238
Atrophic	6 (18.8%)	8 (25.0%)	0.791
Hyperplastic	17 (53.1%)	9 (28.1%)	0.134
Inflammatory infiltrates			
Superficial, perivascular	—	16 (50.0%)	0.001**
Lichenoid/patchy lichenoid	32 (100%)	7 (21.9%)	0.001**
No inflammation	—	9 (28.1%)	0.004**
Fibrosis			
0	6 (18.8%)	6 (18.8%)	1.000
1	22 (68.8%)	22 (68.8%)	1.000
2	3 (9.4%)	3 (9.4%)	1.000
Other changes			
Basal vacuolar degeneration	1 (3.1%)	7 (21.9%)	0.070
Dyskeratotic cells	2 (6.3%)	7 (21.9%)	0.180
Melanophages	8 (25.0%)	23 (71.9%)	0.001**
Vascular changes			
Telangiectasia	7 (21.9%)	14 (43.8%)	0.118
Vascular proliferation	1 (3.1%)	4 (12.5%)	0.250

Mc Nemar test was used. *P< 0.05, **P< 0.01.

**Table 2:** Evaluation of parameters after the treatment, according to response to treatment.

	Response to treatment		
After treatment	Yes	No	*P*
	*n* (%)	*n* (%)	
Epidermotropism			
Single/haloed lymphocytes	3 (13.0%)	4 (44.4%)	0.053
Linearly arranged	2 (8.7%)	4 (44.4%)	0.038*
lymphocytes			
Pautrier microabscesses	1 (4.3%)	3 (33.3%)	0.057
Stratum corneum			
Normal	19 (82.6%)	2 (22.2%)	0.001**
Parakeratosis	0 (0.0%)	1 (11.1%)	0.281
Orthohyperkeratosis	4 (17.4%)	6 (66.7%)	0.007**
Epidermis			
Normal	13 (56.5%)	2 (22.2%)	0.122
Atrophic	7 (30.4%)	1 (11.1%)	0.256
Hyperplastic	3 (13.0%)	6 (66.7%)	0.002**
Inflammatory infiltrates			
Superficial. perivascular	13 (52.1%)	4 (44.4%)	0.433
Lichenoid/patchy lichenoid	2 (8.7%)	5 (55.6%)	0.004**
No inflammation	9 (39.1%)	0 (0%)	0.027*
Fibrosis			
0	6 (26.1%)	0 (0.0%)	0.150
1	15 (65.2%)	7 (77.8%)	0.491
2	1 (4.3%)	2 (22.2%)	0.184
Other changes			
Basal vacuolar degeneration	7 (30.4%)	0 (0.0%)	0.061
Dyskeratotic cells	7 (30.4%)	0 (0.0%)	0.061
Melanophages	18 (78.3%)	5 (55.6%)	0.199
Vascular changes			
Telangiectasia	10 (43.5%)	4 (44.4%)	0.960
Vascular proliferation	3 (13.0%)	1 (11.1%)	1.000

Chi-square and Fisher's Exact tests were used *P < 0.05, **P< 0.01.
